# Large twisted ovarian fibroma in menopausal women: a case report

**DOI:** 10.11604/pamj.2015.20.322.5998

**Published:** 2015-04-06

**Authors:** Majdouline Boujoual, Ihsan Hakimi, Jaouad Kouach, Mohamed Oukabli, Driss Rahali Moussaoui, Mohammed Dehayni

**Affiliations:** 1 Department of Gynecology-Obstetric, University of Medicine Tangier, Military Training Hospital Med V, Rabat, Morocco; 2 Department of Gynecology-Obstetric, Military Training Hospital Med V, Rabat, Morocco; 3 Department of Pathology, Military Training Hospital Med V, Rabat, Morocco

**Keywords:** Ovarian fibroma, torsion, postmenopausal

## Abstract

Ovarian fibroma is the most common benign solid tumors of the ovary, commonly misdiagnosed as uterine fibromaor as malignant ovarian tumors. It occurs generally in older perimenopausal and postmenopausal women. Occasionally large fibromas may undergo torsion causing acute abdominal pain. Doppler Ultrasonographyimaging is the choice study. CT and MRI are often needed for further characterization and differentiation from other solid ovarian masses. The choice treatment is surgical removal with intraoperative frozensection. Immunohistochemicalanalysis is recommended to rule out the differential diagnosis. Here we present a case of a postmenopausal woman with a large twisted ovarian fibroma reflecting diagnostic and management difficulties including potential misdiagnosis of the tumor as a malignant ovarian neoplasm that may influence the surgical approach.

## Introduction

Ovarian fibromas are the most common benign solid tumors of the ovary (1-4%), typically detected in middle aged women [1], often difficult to diagnose preoperatively and commonly misdiagnosed as uterine fibromas, because of their same pathology, complications, clinical and ultrasonic features [2, 3], or sometimes as malignant ovarian tumors because of accompanying ascites and increased serum CA-125 level [4]. Extra uterine fibromas present greater diagnostic challenge. Here we present a case of a 62 year-old postmenopausal with a large twisted ovarian fibroma presented as acute pelvic pain. This case highlights the diagnostic difficulties that may be encountered in the management of twisted ovarian fibroma including potential misdiagnosis of the tumor as a malignant ovarian neoplasm that may influence the surgical approach.

## Patient and observation

**Figure 1:**
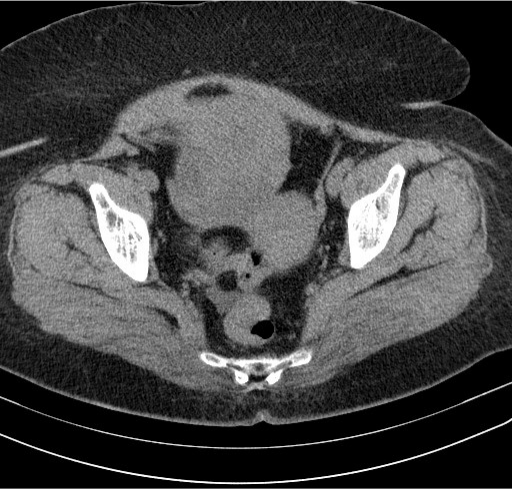
Pelvic computed tomography revealing an enlarged ovary with iso-dense mass in the midline, measuring 122× 86cm with deviation of the uterus

**Figure 2:**
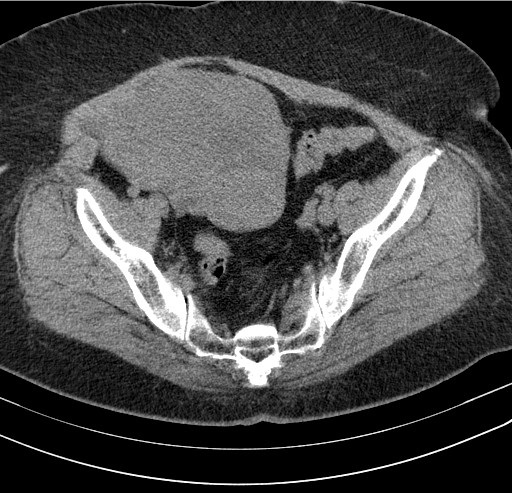
Pelvic computed tomography showing rotated mass toward the contralateral side of the pelviswithout lymphadenopathy or pelvic effusion

**Figure 3:**
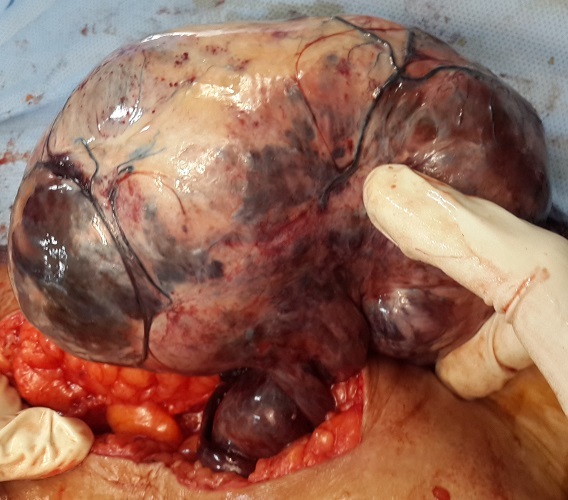
Intraoperative picture showing a black bluish encapsulated mass arising from the twisted right adnexa, measuring 140x 100x 60 mm

**Figure 4:**
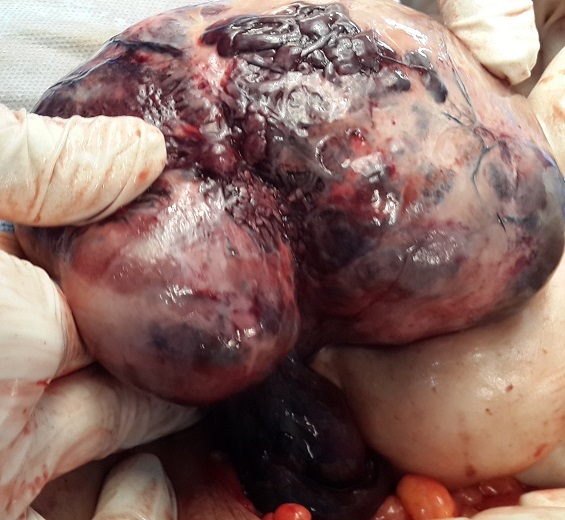
Intraoperative picture showing an ovarian mass with irregular surface, hemorrhagicreshuffle and thrice twisted pedicle

**Figure 5:**
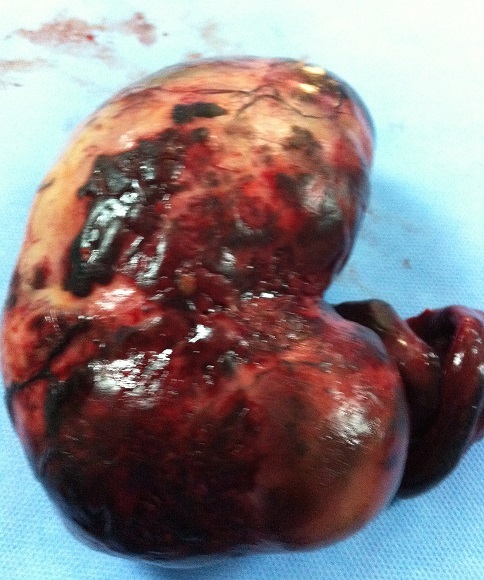
Macroscopic appearance of the resected ovarian fibroma.

**Figure 6:**
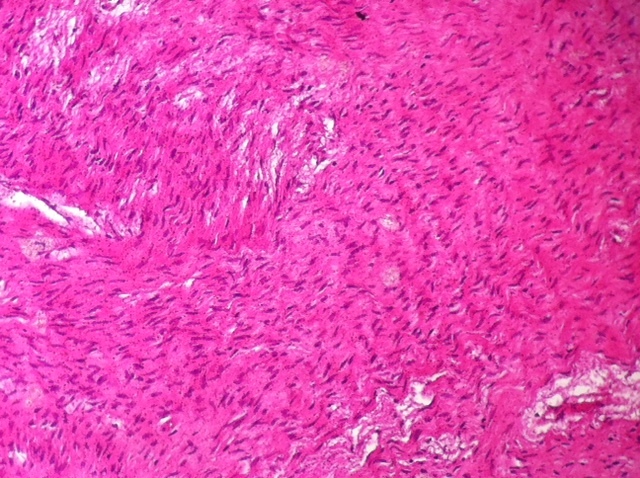
Histopathology of ovarian fibroma showing benignspindle cellproliferation (X4)

**Figure 7:**
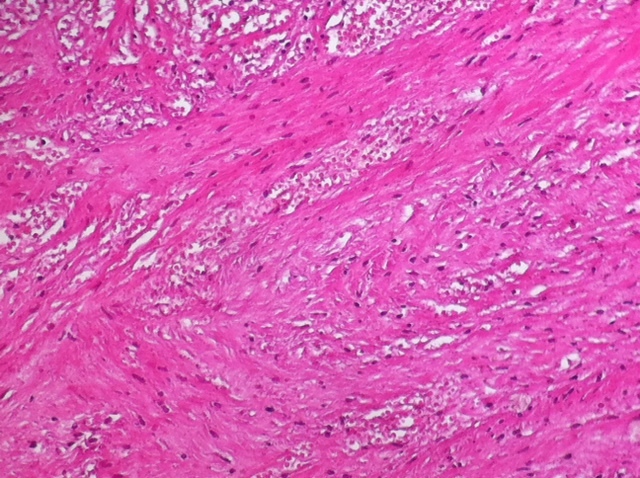
Histopathology of ovarian fibroma showing ischemic necrosis (X4)

A 62-year-old post-menopausal, multiparous woman waspresented in emergency with nausea; vomiting and a 6-day history of worsening abdominal pain started in the right lower quadrant and subsequently spread to the whole abdomen. In her past medical history wereintermittent episodes of abdominal discomfort and a sensation of abdominal heaviness during the preceding months. Her vital signs were all within normal limits. Physical examination revealed a palpable abdominal mass in the lower abdomen with sensibility and involuntary guarding. Vaginal examination revealed a normal sized uterus and a large painful irregular mass of 10 cm size, firm in consistency. Pelvic ultrasonography showed a non-homogeneous mass (120 × 10 mm) in the upper and right latero uterinewithout any flow on color Doppler. Pelvic computed tomography revealed a right enlarged ovary with heterogeneous iso-dense mass in the midline, rotated toward the contralateral side of the pelvis measuring 122× 86cm with deviation of the uterus without lymphadenopathy or pelvic effusion (Figure 1, Figure 2). Anexploratory laparotomy had showed a black bluish encapsulated mass arising from the twisted right adnexa, measuring 140x100x60 mm with irregular surface and hemorrhagicreshuffle, attached to the right ovary with a thrice twisted pedicle (Figure 3, Figure 4, Figure 5). Both the uterus and left adnexa appeared normal. A total hysterectomy and bilateral salpingo-ooferectomy were performed. According to pathological and immunohistochimical findings, the diagnosis of ovarian fibroma with extensive ischemic necrosis was confirmed (Figure 6, Figure 7).

## Discussion

Mesenchymal neoplasms of the ovary are uncommon, not specific and determine difficult problems in diagnosis, histogenesis, behavior, and therapy [5]. Ovarian fibroma is seen between 20 and 65 years oldwith mean ages in the fifth and sixth decades [6–8].

Ovarian fibroma can be bilateral in 4-8% of patients and multiple in 10% of cases [5], especially in Gorlin syndrome [9, 10], or associated with pleural effusion and ascites in Meigs’ syndrome [11]. It is often seen concomitantly with uterine leiomyoma suggesting an identical hormonal stimulation [12] and rarely associated with hirsutism or infertility [13].

These tumors are often asymptomatic despite their large size and are mostly discovered on routine examination [1]. Sometimes, they manifest with abdominal enlargement, urinary symptoms, abdominal pain [5], and occasionally withtorsion [1] which is rare in the postmenopausal patient such us our case [14, 15]. Also, ovarian fibromacan mimic ovarian cancer because of his solid nature, his association with ascites, pleural effusions [16] and elevated CA-125 levels [8] which is more pronounced in torsion due to necrosis and inflammation [4].

Ultrasound features are usually nonspecific [16]. Doppler imaging is the study ofchoice when ovarian torsion is suspected. Classically, the ovary appears enlarged, amorphous, and hypoechoic with heterogeneous stroma because of hemorrhage and edema [15] and coexistent mass, free pelvic fluid, lack of arterial or venous flow, and a twisted vascular pedicle [14].

On CT, ovarian fibroma usually appears as homogeneous solid tumors with delayed enhancement [8, 17]. While, diagnostic criteria for torsion include an adnexal mass in the midline, rotated toward the contralateral side of the pelvis; deviation of the uterus to the side of the affected ovary and ascites [14].

MRI is often needed for further characterization and differentiation of ovarian fibromas from other solid ovarian masses [16]. The tumor appeared as well-circumscribed low signal intensity mass on T1, with mixed signal intensity on T2 due to degeneration of the leiomyoma [12]: hemorrhage, necrosis, cyst formation, calcareous and sarcomatous degeneration [2, 5]. In fact, suggestive signs of malignancy include: solid mass, size greater than 10 cm, internal hypervascularity, advanced age, extension of the tumor into the pelvis or surrounding viscera and metastases [13]. While, suggestive signs of torsion include: tube thickening, ascites, deviation to the twisted side, hemorrhage in the thickened tube, and torsion knot [17].

Because of its rarity, the immunohistochemical analysis with desmin, inhibin α-smooth muscle actin or histochemical staining with Masson′s trichrome is recommended to rule out the differential diagnosis especially leiomyosarcoma and sex-cord stromal tumors (thecoma and sclerosing stromal tumor) [12].

Early recognition of ovarian torsion and restoration of blood flow are important to avoid irreversible ovarian damage. Adnexectomy has been the standard treatment, rather than untwisting the affected ovary due to fear of thromboembolism into the systemic circulation after untwisting the ovarian vasculature [14]. In fact, treatment for ovarian fibroma need surgical removal with intraoperative frozen whether laparoscopic or open. However, surgeons are reluctant to use laparoscopic approach because of extraction difficulty. Moreover, the benign nature cannot be definitely diagnosed preoperatively and safe removal must be achieved without peritoneal contamination [3, 13]. Cystectomy only can be performed in young women [3, 7]. While, a total hysterectomy and bilateral salpingooophorectomy is be the treatment of choice in the elderly patient [13].

## Conclusion

Ovarian fibromas are uncommon but are the most common benign solid tumor of the ovary. Despite its rarity, it should be preoperatively considered in the differential diagnosis. Its treatment requires surgical removal with intraoperative frozen section and immunohistochemical analysis for definitive diagnosis.
